# Event-related potentials and biomarkers of psychiatric diseases: the necessity to adopt and develop multi-site guidelines

**DOI:** 10.3389/fnbeh.2014.00428

**Published:** 2014-12-10

**Authors:** Salvatore Campanella, Cécile Colin

**Affiliations:** ^1^Laboratoire de Psychologie Médicale et d'Addictologie, ULB Neuroscience Institute, CHU Brugmann-Université Libre de BruxellesBrussels, Belgium; ^2^Center for Research in Cognition and Neurosciences, ULB Neuroscience Institute and Laboratoire de Neurophysiologie Sensorielle et Cognitive, CHU Brugmann-Université Libre de BruxellesBrussels, Belgium

**Keywords:** event-related potentials, mismatch negativity, reference electrode, biomarker, psychiatry

Higuchi et al. (2014) published a paper comparing healthy controls, schizophrenic patients and subjects with at-risk mental state (ARMS) during an event-related potential (ERP) auditory oddball task. Results showed that ARMS subjects who transitioned to schizophrenia (Converters) disclosed diminished duration mismatch negativity amplitudes (dMMN) as compared with non-Converters before onset of illness. As the reduction of dMMN is a common finding when schizophrenic patients are compared with healthy controls (e.g., Umbricht and Krljes, 2005), the authors concluded that decreased dMMN amplitudes are a trait biomarker of psychosis, present before and after the development of the disease.

Nowadays, relapse rate for several psychiatric disorders remains tremendously high, suggesting a treatment gap in mental health care (Kohn et al., 2004). Therefore, in psychiatry, there is a recognized need for alternatives to psychotherapy and medication (Dobson et al., 2008). In this view, the identification of biomarkers is important to discover the biological underpinnings of a psychiatric syndrome and could assist in predicting the course of a mental illness in an individual and in tailoring treatment (Singh and Rose, 2009). Higuchi and colleagues' study (2014) is then of the greatest relevance, and the authors have to be commended for their work. Nevertheless, if we totally caution the use of ERPs as biomarkers of mental diseases (Campanella, 2013), we also would like to stress the urgent need to develop and promote multisite guidelines to record electrophysiological measures that may be compared and used across studies. Some guidelines concerning main ERP components obviously exist (e.g., Duncan et al., 2009, for MMN, P300 and N400). However, their use across studies is still not guaranteed, and this may lead, as in Higuchi and colleagues' study, to some misinterpretations of the data.

By employing duration changes in repetitive background stimulation while the patient was reading or watching videos, dMMN amplitude was found to be attenuated in several pathological disorders (for a review, Näätänen et al., 2012). According to the dominant view, MMN is the outcome of an automatic, *attention-independent*, comparison process between a deviant stimulus and the memory trace formed by the sensory representation of the standard stimulus within short-term memory (Novak et al., 1990). A major clinical interest of the MMN therefore consists in recording this brain activity *outside the focus of attention* of the participant, to monitor the detection of any change in ongoing auditory stimulation, irrespective of where attention is directed (Duncan et al., 2009). This is of particular interest in patients with severe brain injuries or in persistent vegetative/minimal consciousness state (Kotchoubey et al., 2003), but it also triggers a crucial problem in “conscious” patients, which is to ensure that no *active attention* was directed to the task. Indeed, when participants selectively attend to deviant stimulations, a negativity of central cortical distribution (seen only during conscious stimulus attention), called N2b, superimposed the MMN (Patel and Azzam, 2005). Overall, auditory oddball tasks involved two negative overlapping sub-components evoked around 200 ms: the MMN and the N2b, elicited in passive vs. active conditions, and representing automatic vs. conscious processes, respectively (Naatanen et al., 2002). One main “technical” point allows ensuring that a passive auditory oddball task has generated a “pure” MMN, not contaminated by active (conscious) N2b attentional processes: MMN exhibits a phase reversal (i.e., positive polarity) over mastoid and other lateral posterior sites over the same latency range when a nose reference is used (e.g., Alho, 1995; Grimm et al., 2004), while N2b does not (e.g., Aulanko et al., 1993; Sussman et al., 2002).

This phase reversal, representing the only mean to disentangle MMN from N2b activities (see Figure 1 for illustration), has been largely replicated in studies from our laboratory (e.g., Colin et al., 2009) as well as from other research centers (see Näätänen et al., 2007 for a review). Unfortunately, this point is missing in Higuchi and collaborators' study, as electrodes were referred to the average amplitude of the ear electrodes (Higuchi et al., 2014). This clearly does not affect the conclusion that their recorded negativity allowed separating Converters from non-Converters before the onset of illness; however, it clearly question whether it is *MMN or N2b* amplitude reduction that should be considered as a biomarker of psychosis. As these sub-components refer to different (passive vs. active) attentional processes, this interpretation is of the greatest clinical relevance, because alterations of covert vs. overt attention processes would involve specific cognitive rehabilitative programs (Kim et al., 2009). Moreover, even if this point is minor in the paper, the authors also reported that “P3a amplitudes were barely detectable” in their study (Higuchi et al., 2014). Here again the explanation could be “technical,” as P3a component is usually recorded through a three-stimulus oddball task, in which “novel” events (e.g., dog barks), presented as infrequent distractors in a train of more “typical” target and standard stimuli (e.g., tones), will generate a fronto-central P3a, whereas the infrequent target stimuli will elicit a parietal P3b (see Polich, 2007 for a review). However, Higuchi et al. (2014) only used a standard/target auditory oddball task, without distractor stimuli, which is not the best suited paradigm to generate P3a in passive oddball tasks (Jeon and Polich, 2001).

**Figure 1:**
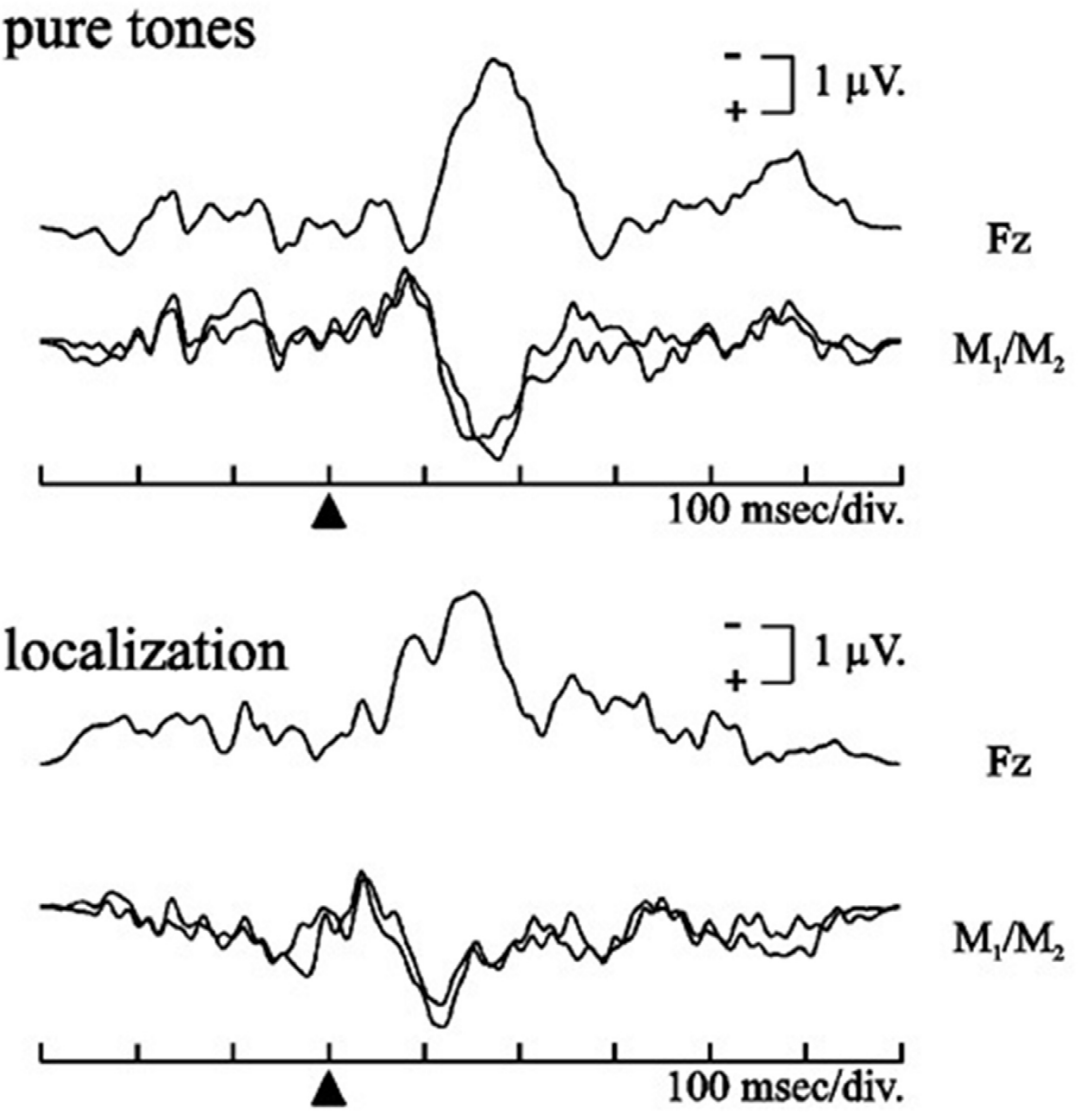
**Differential waveforms obtained in auditory oddball paradigms using a nose reference for a frequency contrast with pure tones (upper part) and for a localization contrast with syllables (lower part)**. The black triangle indicates sound onset. Polarity reversal between Fz and both mastoid (M1/M2) is clearly exhibited (adapted with permission from Colin et al., 2002).

To conclude, we totally endorse the main objective of Higuchi and collaborators' study, which was to investigate whether a neurophysiological biomarker of psychosis onset may be found in ARMS subjects. Given their state independence, such biomarkers hold much promise in prevention research because they can be used to identify people who are at high risk of developing psychiatric disorders (Beauchaine, 2009). However, reported data also stressed that several challenges must be overcome before ERPs gain widespread use as biomarkers in psychiatry (Luck et al., 2011). Among these, the promotion of multi-site guidelines to record electrophysiological measures, notably within high-density ERP datasets (Murray et al., 2008), that may be compared and used across studies is urgent, as this could help to avoid functional misinterpretations of the data as well as to prevent from the emergence of controversial results from different laboratories.

## Funding

The first author is funded by the Belgian Fund for Scientific Research (F.N.R.S., Belgium), but this fund did not exert any editorial direction or censorship on any part of this article.

### Conflict of interest statement

The authors declare that the research was conducted in the absence of any commercial or financial relationships that could be construed as a potential conflict of interest.
